# Histiocytoid cardiomyopathy and microphthalmia with linear skin defects syndrome: phenotypes linked by truncating variants in *NDUFB11*

**DOI:** 10.1101/mcs.a001271

**Published:** 2017-01

**Authors:** Gillian Rea, Tessa Homfray, Jan Till, Ferran Roses-Noguer, Rachel J. Buchan, Sam Wilkinson, Alicja Wilk, Roddy Walsh, Shibu John, Shane McKee, Fiona J. Stewart, Victoria Murday, Robert W. Taylor, Michael Ashworth, A. John Baksi, Piers Daubeney, Sanjay Prasad, Paul J.R. Barton, Stuart A. Cook, James S. Ware

**Affiliations:** 1 NIHR Cardiovascular Biomedical Research Unit at Royal Brompton and Harefield NHS Foundation Trust and Imperial College London, London SW3 6NP, United Kingdom;; 2 National Heart and Lung Institute, Imperial College London, London SW3 6NP, United Kingdom;; 3 Northern Ireland Regional Genetics Service, Belfast City Hospital, Belfast, BT9 7AB, United Kingdom;; 4 Royal Brompton and Harefield NHS Foundation Trust, London SW3 6NP, United Kingdom;; 5 Department of Clinical Genetics, Laboratory Medicine, The Queen Elizabeth University Hospital, Glasgow G51 4TF, United Kingdom;; 6 Wellcome Trust Centre for Mitochondrial Research, Institute of Neuroscience, Newcastle University, Newcastle upon Tyne, NE2 4HH, United Kingdom;; 7 Histopathology Department, Camelia Botnar Laboratories, Great Ormond Street Hospital for Children NHS Trust, London WC1N 3JH, United Kingdom;; 8 MRC Clinical Sciences Centre, Imperial College London, London W12 0NN, United Kingdom;; 9 National Heart Centre Singapore, Singapore 169609, Singapore

**Keywords:** asymmetric, linear skin defects, dilated cardiomyopathy, left ventricular noncompaction cardiomyopathy, linear hyperpigmentation, microphthalmos, oncocytic cardiomyopathy, ventricular fibrillation, Wolff–Parkinson–White syndrome

## Abstract

Variants in *NDUFB11,* which encodes a structural component of complex I of the mitochondrial respiratory chain (MRC), were recently independently reported to cause histiocytoid cardiomyopathy (histiocytoid CM) and microphthalmia with linear skin defects syndrome (MLS syndrome). Here we report an additional case of histiocytoid CM, which carries a de novo nonsense variant in *NDUFB11* (ENST00000276062.8: c.262C > T; p.[Arg88*]) identified using whole-exome sequencing (WES) of a family trio. An identical variant has been previously reported in association with MLS syndrome. The case we describe here lacked the diagnostic features of MLS syndrome, but a detailed clinical comparison of the two cases revealed significant phenotypic overlap. Heterozygous variants in *HCCS* (which encodes an important mitochondrially targeted protein) and *COX7B,* which, like *NDUFB11,* encodes a protein of the MRC, have also previously been identified in MLS syndrome including a case with features of both MLS syndrome and histiocytoid CM. However, a systematic review of WES data from previously published histiocytoid CM cases, alongside four additional cases presented here for the first time, did not identify any variants in these genes. We conclude that *NDUFB11* variants play a role in the pathogenesis of both histiocytoid CM and MLS and that these disorders are allelic (genetically related).

## INTRODUCTION

*NDUFB11* (OMIM *300403) variants have recently been independently proposed to cause histiocytoid cardiomyopathy (histiocytoid CM; OMIM 500000) (Shehata et al. 2015) and microphthalmia with linear skin defects syndrome (MLS; OMIM 309801) (van Rahden et al. 2015). Histiocytoid CM is a rare, distinctive form of cardiomyopathy with approximately 150 cases reported worldwide, it has numerous synonyms including oncocytic cardiomyopathy (Shehata et al. 2011). Predominantly affecting females early in life, it is characterized by arrhythmias and associated sudden death (Gilbert-Barness and Barness 2006). Associated cardiac abnormalities include ventricular and atrial septal defects, endocardial fibroelastosis, and hypoplastic left heart syndrome (Shehata et al. 1998). Extracardiac features involving the nervous system and eyes are also frequently reported (Malhotra et al. 1994). Variants in mitochondrial DNA (mtDNA) have previously been associated with histiocytoid CM (Vallance et al. 2004), but this has not been replicated in further studies. Histological findings in cardiac tissue often show an accumulation of excessive, aberrantly shaped mitochondria, supporting the role of mitochondrial dysfunction in the etiology (Shehata et al. 2015).

MLS syndrome, also known as MIDAS syndrome (microphthalmia, dermal aplasia, and sclerocornea) (Happle et al. 1993), was first described in 1988 (Al-Gazali et al. 1988). It is a rare X-linked disorder with male lethality in utero*,* characterized by unilateral or bilateral microphthalmia and linear skin defects. Skin defects are classically limited to the face and neck along Blaschko's lines, are present from birth, and heal with time, often leaving minimal scarring. Additional clinical features may include neurological and cardiac abnormalities (Morleo and Franco 1993). Heterozygous variants in the X-encoded genes *HCCS* (OMIM *300056) (Wimplinger et al. 2006), which encodes an important mitochondrially targeted protein, and *COX7B* (OMIM *300885) (Indrieri et al. 2012), which encodes a component of the mitochondrial respiratory chain (MRC), have been identified previously in MLS-affected females (Indrieri et al. 2012). However, variants are not detected in all cases, suggesting genetic heterogeneity.

More than 20 years ago, Bird and coworkers reported an infant with the characteristic skin lesions of the then newly recognized MLS syndrome and a normal 46, XX karyotype, who died suddenly and unexpectedly at 4 mo of age. Death was attributed to oncocytic (histiocytoid) cardiomyopathy (Bird et al. 1994). The authors noted that the “coexistence of two rare conditions, one of which mapped to the X Chromosome, and an excess of affected females with oncocytic cardiomyopathy, make it likely that oncocytic cardiomyopathy is also X-linked, with Xp22 being a candidate region. Overlapping manifestations in the two conditions (ocular abnormalities in cases of oncocytic cardiomyopathy and arrhythmias in MLS) offer additional support for this hypothesis” (Bird et al. 1994). Here, we present evidence in support of the allelic nature of these conditions. We describe a new case of histiocytoid CM in whom we identified a de novo nonsense variant in *NDUFB11* (c.262C > T; p.(Arg88*)) (see Table 1). An identical variant has recently been reported as the molecular basis of a case of MLS syndrome. We compare the clinical features of these cases (see Table 2), highlighting that although each of the two entities show distinct manifestations, they share substantial phenotypic overlap and potential mechanisms are outlined.

**Table 1.** Summary of variant of interest in *NDUFB11*

HGNC symbol	*NDUFB11*
Name	NADH:ubiquinone oxidoreductase subunit B11
Genomic location GRCh37 (hg19)	Chromosome X: 47002089
HGVS cDNA	ENST00000276062.8 c.262C > T
HGVS protein	p.(Arg88*)
Predicted effect	Stop gained
Genotype	Heterozygous
Inheritance mode	Sporadic
Frequency in ExAC	0

HGNC, HUGO Gene Nomenclature Committee; HGVS, Human Genome Variation Society; ExAC, Exome Aggregation Consortium.

**Table 2.** Detailed comparison of the clinical features of two individuals with the same *NDUFB11* truncating variant (ENST00000276062.8: c.262C > T; p.(Arg88*))—one manifesting primarily as histiocytoid CM, the other with microphthalmia and linear skin defects syndrome

Phenotypic feature	Case described here (Case 1)	Case reported by van Rahden et al. 2015
Presenting phenotype	Histiocytoid CM	Microphthalmia and linear skin defects syndrome
Antenatal history	Healthy, nonconsanguineous parents	Healthy, nonconsanguineous parents
	Alternating bradycardia and tachycardia noted at 27 wk; emergency Cesarean section at 38 wk for fetal tachycardia
Sex	Female	Female
Birth weight	3690 g (91st centile)	3060 g (10th–25th centile)
Cardiac arrhythmias	Neonatal episodes of supraventricular tachycardia; collapse with first documented ventricular tachycardia (VT) at 7 mo; continued VT “storms” necessitating drug treatment, dual chamber implantable cardiac defibrillator, and left thoracic sympathectomy; cardiac transplantation was carried out at 13 mo	Sudden unexpected cardiac arrest aged 6 mo; repeated treatment for ventricular arrhythmias; death within several weeks
Cardiomyopathy	Features of left ventricular noncompaction; preserved systolic function	Nil reported
Cardiac histology	Histiocytoid CM	Histiocytoid CM
Eye abnormalities Microphthalmia Scleroderma Other eye abnormalities	Not present Not present Intermittent squint	Nil reported Nil reported Lacrymal duct atresia
Neuromuscular	Mild-to-moderate bulbar palsy	Axial hypotonia present from birth
Skin abnormalities	None present	Linear skin defects on nose, chin, and neck present at birth, disappeared in the first few months of life
Thyroid abnormalities	Focal histiocytoid change in the thyroid (and also lungs and choroid plexus of the brain)	Oncocytic metaplasia evidence on postmortem
Other anomalies	Severe feeding difficulties gastroesophageal reflux, requiring Nissen fundoplication and percutaneous endoscopic gastrostomy	Failure to thrive documented from 1 mo of age
Evidence of somatic mosaicism	No evidence in DNA from lymphocytes	Yes, in DNA from lymphocytes and fibroblasts

CM, cardiomyopathy.

## RESULTS

**Figure 1.**
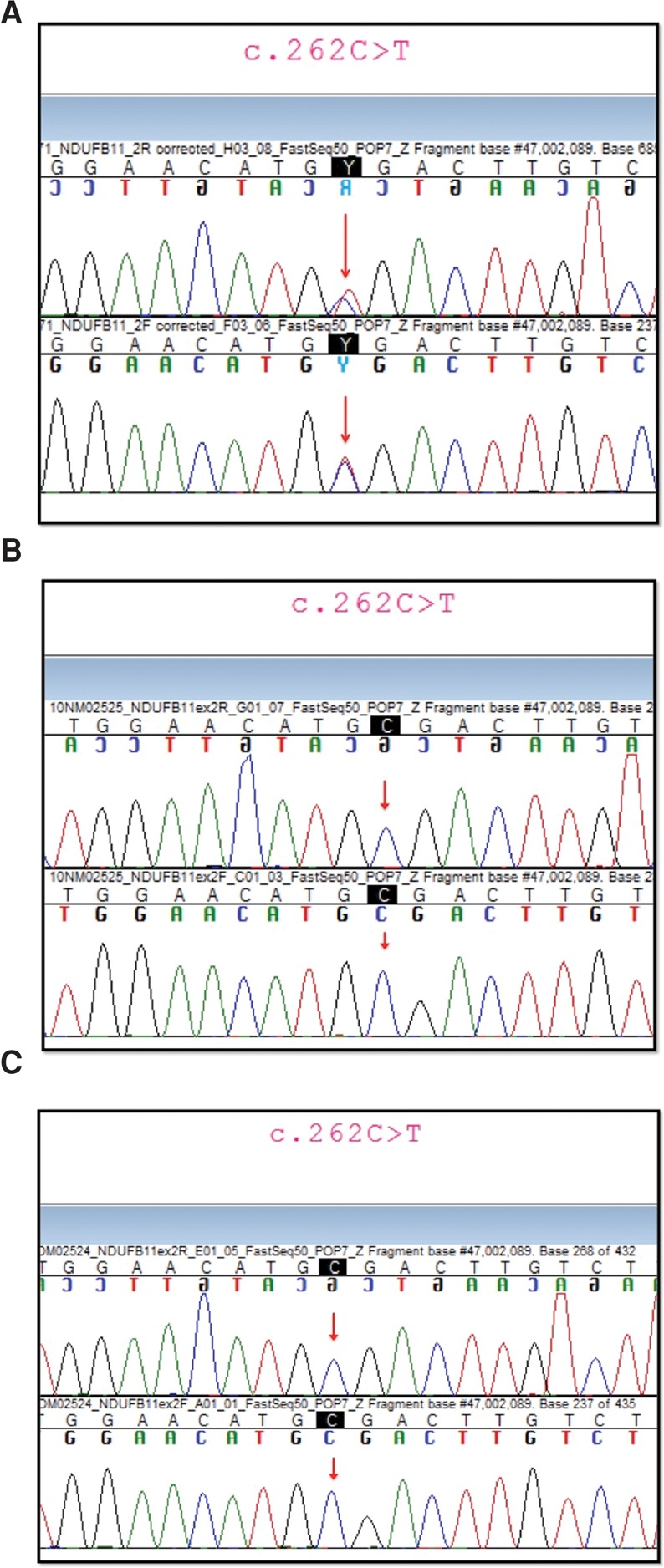
(*A*) Sequence electropherogram from genomic DNA of child affected by histiocytoid cardiomyopathy (CM) from the family trio showing the *NDUFB11* variant identified by whole-exome sequencing (WES) and confirmed by Sanger sequencing. The image shows part of *NDUFB11* exon 2. Forward and reverse reads are shown. The red arrows point to the double peak in the electropherogram showing heterozygosity for the nonsense variant in *NDUFB11* c.262C > T. The next-generation sequencing (NGS) coverage at this base was 62×, with 27 reference reads and 33 alternate reads. (*B*) Sequence electropherogram from genomic DNA of the unaffected mother from the trio showing no evidence of the *NDUFB11* sequence-level variant that had been identified in the affected child. The image shows part of *NDUFB11* exon 2. Forward and reverse reads are shown. The red arrows point to the base where the nonsense variant in *NDUFB11* c.262C > T was detected in the affected daughter. The coverage at this base on WES was 50×, with no evidence of the variant. (*C*) Sequence electropherogram from genomic DNA of the unaffected father from the trio showing no evidence of the *NDUFB11* variant that was identified in the affected child. The image shows part of *NDUFB11* exon 2. Forward and reverse reads shown. The red arrows point to the base where the nonsense variant in *NDUFB11* c.262C > T was detected in the affected daughter. The coverage at this base on WES was 26×, with no evidence of the variant.

The trio sequencing approach identified two rare de novo variants, predicted to be protein altering in the proband: *NDUFB11* c.262C > T; p.(Arg88*) and *FAM135A* c.474C > G; p.(Tyr158*). Sanger sequencing confirmed the presence of both variants in the affected child and their absence in the unaffected parents (see Fig. 1). *NDUFB11* was a highly plausible candidate gene, located on the X Chromosome, providing a potential mechanism for the predominance of females affected (with presumed in utero male lethality), with a mitochondrial function in keeping with previous understanding of histiocytoid CM as a mitochondrial disorder. Alternative inheritance models (monoallelic or biallelic) were considered with no additional strong candidate genes identified. We interrogated four additional unrelated cases of histiocytoid CM for variants in *NDUFB11*, C*OX7B*, or *HCCS*, and no putative disease-causing variation was identified. We also reviewed publicly available WES data (Shehata et al. 2015) from three additional cases of histiocytoid CM without *NDUFB11* variants and found no evidence of de novo or rare variants in either *HCCS* or *COX7B*.

## DISCUSSION

**Figure 2.**
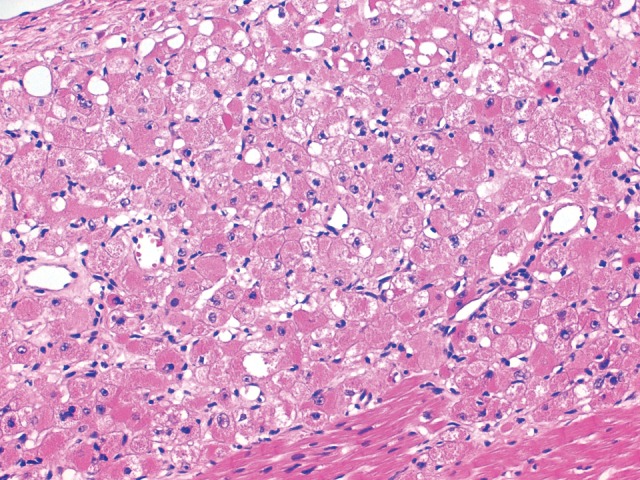
Histology of histiocytoid cardiomyopathy. Longitudinal section of myocardium from an infant with histiocytoid cardiomyopathy. A few normal myocytes are present in the *lower* part of the field. The remaining cells are histiocytoid myocytes.

At the outset of our study the molecular etiology of histiocytoid CM was unknown; we undertook WES in a trio involving an affected child (see Fig. 2) and both unaffected parents. This identified a de novo nonsense variant in *NDUFB11,* an X-encoded mitochondrial gene, consistent with the suspected role of mitochondrial dysfunction in histiocytoid CM (Ferrans et al. 1976) and a de novo nonsense variant in *FAM135A*.

Simultaneous to our analyses, two independent reports showed evidence of variants in *NDUFB11* associated with histiocytoid CM (Shehata et al. 2015) and MLS syndrome (van Rahden et al. 2015). The phenotypic associations of these additional variants in *NDUFB11* and ours are shown in Table 3. An identical variant to that reported here has recently been reported in a case of MLS in whom histiocytoid CM was found postmortem (van Rahden et al. 2015) indicating a shared molecular basis for these conditions. Our case lacked the diagnostic features of MLS syndrome (see Table 2). Although *FAM135A* was not formally excluded as a candidate, there was no additional evidence to implicate a role for it in histiocytoid CM.

**Table 3.** Catalog of variants reported in *NDUFB11,* with associated phenotypes

	Case 1	Case 2	Case 3^a^	Case 4^a^	Case 5^a^	Case 6	Case 7
Reported diagnosis	Histiocytoid CM	MLS syndrome	MLS syndrome	Asymptomatic	Terminated pregnancy at 24th wk gestation	Histiocytoid CM	Histiocytoid CM
Sex	Female	Female	Female	Female	Female	Female	Female
Cardiac phenotype	Histiocytoid CM	Histiocytoid CM	Developed dilated cardiomyopathy at 2 mo of age requiring heart transplant at 6 mo of age^b^	Nil reported	Thickened myocardium Pericardial effusion	Histiocytoid CM	Histiocytoid CM
Eye phenotype	Intermittent squint	Lacrimal duct atresia	Myopia, nystagmus, and strabismus	Nil reported	Nil reported	Nil reported	Nil reported
Skin phenotype	Nil	Linear skin defects on nose, chin, and neck present at birth	Linear and atrophic hyperpigmented streaks on face and neck	Nil reported	Nil reported	Nil reported	Nil reported
Protein variant^c^	p.(Arg88*)	p.(Arg88*)	p.(Arg134Serfs*3)	p.(Arg134Serfs*3)	p.(Arg134Serfs*3)	p.(Trp85*)	p.(Tyr108*)
Genomic coordinate^d^	47002089	47002089	47001806	47001806	47001806	47002097	47002027
Exon	Exon 2	Exon 2	Exon 3	Exon 3	Exon 3	Exon 2	Exon 2
Status	De novo	De novo	Inherited from asymptomatic mother	Unknown	Inherited from asymptomatic mother	De novo	De novo
Other phenotypic features	Bulbar palsy Severe feeding difficulties		Seizures Developmental delay Agenesis of the corpus callosum Severe muscular hypotonia Delayed dentition Growth parameters below the 3rd centile at 15/12		Intra-uterine growth retardation Dysgenesis of the corpus callosum Small cerebellum Connection between a lateral ventricle and the cavum septum pellucidum		
Reference	Case presented here	Subject 1^e^	Subject 2^e^	Mother of Subject 2^e^	Aborted fetus from the mother of subject 2^e^	GHC-G^f^	GHC-T^f^

CM, cardiomyopathy; MLS, microphthalmia with linear skin defects.

^a^Three cases are from a single pedigree and they also have a heterozygous deletion of at least 70 kb at 2p16.3, containing part of *NRXN1,* which may have influenced the phenotype. Intragenic mutations and deletions of *NRXN1* are enriched in cohorts with neurodevelopmental and autistic spectrum disorders. Incomplete penetrance is reported.

^b^No evidence of histiocytoid CM reported, although further review of histology in light of current knowledge would be of interest.

^c^ENST00000276062.8.

^d^GRCh37(hg19).

^e^van Rahden et al. 2015.

^f^Shehata et al. 2015.

Seventy five percent of cases of histiocytoid CM present in female infants (Shehata et al. 2015), often below 2 yr of age (Gilbert-Barness and Barness 2006), and the identification of *NDUFB11*, found on Xp11.23, as an underlying cause explains the large excess of affected females. There have been no molecular reports yet to confirm this as a cause of embryonic male lethality, although Shehata and coworkers comment it is “tempting to speculate that similar mutations occurring in males are embryonic lethal and cause miscarriage, since there would be no residual protein activity” (Shehata et al. 2015).

There is evidence for genetic heterogeneity in histiocytoid CM, as variants in *NDUFB11* and other candidate genes described here do not explain all cases. We hypothesize that *HCCS* (OMIM 30056) and *COX7B* (OMIM 300885)*,* which are X-linked genes encoding or targeting mitochondrial proteins and are implicated in MLS, are good candidate genes for histiocytoid CM. We note that van Rahden et al. (2014) have reported previously a female child with a de novo *HCCS* nonsense variant (c.589C > T; p.(R197*)) and extremely skewed X-inactivation (98:2) with classical features of MLS including linear skin defects on her neck, microphthalmia, anophthalmia, and sclerocornea, who died at 4 mo with ventricular tachycardia (VT) and was found to have histiocytoid CM on postmortem examination, further supporting that MLS and histiocytoid CM are allelic disorders. However, no rare variants were found in *HCCS* or *COX7B* in seven additional histiocytoid CM cases without *NDUFB11* variants (four described here, three in Shehata et al. 2015), though this small series does not exclude a role in “isolated” histiocytoid CM.

Comparisons (see Table 2) between the phenotype in the case of histiocytoid CM presented here and the case of MLS with an identical *NDUFB11* variant (van Rahden et al. 2015) show considerable phenotypic overlap. The case of MLS also had histiocytoid CM; a feature only identified on postmortem examination. The vast majority of cases of histiocytoid CM are identified postmortem or on examination of the native explanted heart during cardiac transplantation. It is possible that additional cases of MLS also have histiocytoid CM but it remains undetected, as has previously been reported (Bird et al. 1994). Previously there was no specific clinical indication to perform detailed cardiac phenotyping in MLS and so further data are lacking. Wimplinger et al. 2006 reported a female, born to healthy nonconsanguineous parents after an uneventful pregnancy, found to have bilateral microphthalmia and sclerocornea but no erythematous skin lesions. She had a de novo *HCCS* nonsense variant (c.589C > T; p.(R197*)) that was also reported in an additional case with both MLS and histiocytoid CM (van Rahden et al. 2014). Around 1 yr of age she was reported to have developed “idiopathic VT” (Wimplinger et al. 2006). In the absence of cardiac histology a diagnosis of histiocytoid CM cannot be confirmed, but we suggest it could underlie her VT.

Wide intra- and interfamilial variation in MLS is recognized including carrier females with variants in *HCCS* or *NDUFB11* (see Table 2) or terminal Xp deletions but no clinical features of MLS syndrome (van Rahden et al. 2015). There are several potential explanations for the wide phenotypic variation including somatic mosaicism; in their case of MLS (see Table 2) van Rahden and coworkers noted that the mutated base thymine was present in 42 sequence reads and the wild-type base cytosine in 84, suggesting that the variant was present in mosaic state in lymphocytes. DNA isolated from fibroblasts was also suggestive of mosaicism (van Rahden et al. 2015). In addition, Shehata et al. (2015) note that in one of their cases with a nonsense *NDUFB11* variant (case GHCG) a second nonsense variant was detected in cytochrome *b*, but only at a frequency of 20% and only in the cardiac tissue, possibly indicating a clonal selection of a somatic variant in the diseased heart. The authors suggest this is one of the potential avenues through which the expressivity of defects in MRC complex 1 activity might be regulated (Shehata et al. 2015). X-inactivation may also influence phenotypic variability as the majority of MLS-affected females have severe skewing of X Chromosome inactivation suggesting that variants in causative X-linked gene(s) cause selective loss of cells in which the mutated X Chromosome is active (van Rahden et al. 2015). Other causes of phenotypic variability could include copy-number variation or common or rare modifier variants contributing to mitochondrial function (Shehata et al. 2015). Furthermore, phenotypic variability may reflect differing abilities of developing tissues and organs in embryonic cells to handle a defective MRC system (van Rahden et al. 2014).

### Conclusion

We conclude that variants in *NDUFB11* are an important cause of histiocytoid CM and report that histiocytoid CM and MLS, which are genetically heterogeneous, are allelic disorders. However, many histiocytoid CM cases remain unexplained by known genes, suggesting a heterogeneous genetic architecture with further contributing genes remaining to be discovered. Histiocytoid CM typically affects female infants <2 yr of age and the identification of *NDUFB11*, which is found on the chromosome band Xp11.23, as an underlying cause explains the large excess of affected females. Comparison of the phenotypes of histiocytoid CM and MLS shows both considerable overlap and features specific to each entity. Differences in phenotypic expression may be due to patterns of X-inactivation, somatic mosaicism, presence of additional variants, environmental factors, or epigenetic factors. We suggest that individuals with MLS syndrome or variants or cytogenetic abnormalities involving *NDUFB11, HCCS*, or *COX7B* should be considered at risk for histiocytoid CM and screening for evidence of malignant arrhythmias and cardiomyopathy may be appropriate. Additional nuclear encoded or mtDNA genes within the MRC are good candidates for further causes of both histiocytoid CM and MLS syndrome.

## METHODS

### Samples

An infant with histiocytoid CM and both unaffected parents were recruited through the NIHR Cardiovascular Biomedical Research Unit at the Royal Brompton and Harefield NHS Foundation Trust. Four additional biologically unrelated histiocytoid cardiomyopathy probands were recruited via U.K. regional genetics services. Studies were performed according to institutional guidelines, with ethical approval.

### Whole-Exome Sequencing Using a Trio Approach

The trio (of affected child and both unaffected parents) underwent WES using the Agilent SureSelect system (Human All Exon v4 + UTR kit) with Illumina sequencing (HiSeq 2500, 100-bp paired-end). Reads were aligned to the human reference genome (hg19) using Burrows–Wheeler alignment (BWA) (Li and Durbin 2009) v0.7.5, and variants were called using the Genome Analysis Toolkit (GATK) v2.8-1 software package (McKenna et al. 2010) according to GATK Best Practice recommendations (DePristo et al. 2011; Van der Auwera et al. 2013). In the affected child, mother, and father, WES generated 11.9 gigabases (Gb), 9.1 Gb and 10.4 Gb of data, respectively. The percentage callable was 99.4, 97.8, and 99.1, respectively (see Table 4). Variants were prioritized using the open source software platform xBrowse (https://atgu.mgh.harvard.edu/xbrowse). Only variants that passed standard GATK filters, with genotype quality score >20, coverage >20×, and allelic balance >20, were included in the analysis. We filtered for rare (minor allele frequency [MAF] <0.001 in Exome Aggregation Consortium [ExAC], 1000 Genomes, and xBrowse reference samples) de novo variants, predicted to be protein altering (nonsense, frameshift, essential splice site, missense, and in-frame insertion or deletion) in the proband. All inheritance models were considered.

**Table 4.** Summary figures of coverage for WES in each sample of the family trio

Sample	Total reads	Mapped reads	Percentage mapped	Percentage on target	Bases 10 ×	Bases 20 ×	Bases 30 ×	Percentage callable	Mean coverage	Median coverage
Affected child	11,906,7512	118,785,553	99.8	81.7	97.8	93.4	87.6	99.4	113	89
Unaffected father	91,775,652	91,571,480	99.8	69.4	91.9	80.5	70.0	97.8	74	53
Unaffected mother	103,827,148	103,582,562	99.8	82.8	98.7	96.5	90.2	99.1	101	77

The trio consists of a child affected with histiocytoid cardiomyopathy and both unaffected parents. Figures are for the coverage of the protein-coding target. Sequencing was undertaken using SureSelect Human All Exon V4 + UTR design (71 Mb) and the Illumina HiSeq 2500 platform. WES, whole-exome sequencing.

### Sanger Sequencing of *NDUFB11*

The coding exons and intron–exon boundaries of *NDUFB11* were amplified by polymerase chain reaction (PCR), and directly sequenced using the BigDye Terminator Cycle Sequencing Kit and an ABI3500 Genetic Analyzer (Applied Biosystems). Sequences were analyzed using Sequencher (v5.3) software. The coding sequence of all samples was fully covered in both directions.

### Sequencing Primers

The sequencing primers were, for *NDUFB11*, F-TCCAGCCATGACTAGAGCTG, R-TCATC TCAGCTCCCCATTCC, and for *FAM135A*, F-GTTGTACTGCAGCCTTGTAATAAAC, R-CAGCCTGAAGAACCATGACC.

## ADDITIONAL INFORMATION

### Data Deposition and Access

*NDUFB11* (SCV000297804.1) and *FAM135A* (SCV000297805.1) variants have been submitted to ClinVar (htpp://www.ncbi.nlm.nih.gov/clinvar/). We do not have consent from patients to deposit complete sequencing data in a repository.

### Ethics Statement

This study received research ethics committee approval (UK NRES Committee South Central—Hampshire B, reference 09/H0504/104) and all subjects (or parents where appropriate) provided written informed consent.

### Acknowledgments

We thank the families presented in this report for their participation.

### Author Contributions

G.R., P.J.R.B., S.A.C., and J.S.W. conceived and designed the study; G.R., J.T., F.R., T.H., M.A., R.J.B., A.W., S.W., S.J., and R.W. collected and analyzed data. G.R., P.J.R.B., S.A.C., and J.S.W. interpreted the data and wrote the paper. All authors have reviewed and approved the final manuscript.

### Funding

This work was supported by the Academy of Medical Sciences, Wellcome Trust, British Heart Foundation, Arthritis Research UK, National Institute for Health Research (NIHR) Biomedical Research Unit in Cardiovascular Disease at Royal Brompton and Harefield NHS Foundation Trust and Imperial College London, Medical Research Council (UK), Foundation Leducq, a Health Innovation Challenge Fund (HICF) award from Wellcome Trust and Department of Health, The Lily Foundation, and the UK NHS Highly Specialised Commissioners.

### Competing Interest Statement

The authors have declared no competing interest.

### Referees

Gholson J. Lyon

Bahig M. Shehata
